# Three new species of *Marasmius* (Agaricales, Basidiomycota) from southwestern China

**DOI:** 10.3897/mycokeys.136.196875

**Published:** 2026-07-15

**Authors:** Chun-Ying Deng, Tai-Shun Li, Zhun Xiang, Qi Zhao

**Affiliations:** 1 Guizhou Institute of Biology, Guizhou Academy of Sciences, Guiyang 550009, China State Key Laboratory of Phytochemistry and Natural Medicines, Yunnan Key Laboratory for Fungal Diversity and Green Development, Kunming Institute of Botany, Chinese Academy of Sciences Kunming China; 2 Guizhou Provincial Key Laboratory of Agricultural Microbiology, Guiyang 550009, China Guizhou Institute of Biology, Guizhou Academy of Sciences Guiyang China; 3 State Key Laboratory of Phytochemistry and Natural Medicines, Yunnan Key Laboratory for Fungal Diversity and Green Development, Kunming Institute of Botany, Chinese Academy of Sciences, Kunming 650201, China Guizhou Provincial Key Laboratory of Agricultural Microbiology Guiyang China

**Keywords:** Marasmiaceae, new taxa, phylogenetic analysis, taxonomy

## Abstract

*Marasmius* is a large and important genus in the forest, with potential research and application value, while its diversity in China remains under-documented. In this work, three new species of *Marasmius* belonging to subgen. *Globulares* were studied from southwestern China. Morphologically, *Marasmius flavosulcatus* is characterized by the lemon-yellow, plicated pileus and large basidiospores. *Marasmius griseobrunneus* is characterized by a white to grayish brown, faintly striate pileus, a stipe tapering towards the base, subdistant lamellae, medium-sized basidiospores (7.06 × 3.91 μm), and clavate caulocystidia. *Marasmius variegatus* is characterized by a yellowish, spotted pileus and dimorphic cells in both the cheilocystidia and the pileipellis. Based on ITS and LSU phylogenetic analyses, the three species belong to the *purpureostriatus*, *wynneae* and *luteoli* series, respectively. In this study, descriptions, photographs of the basidiomata, line drawings, phylogenetic analysis results and comparisons with related species are provided.

## Introduction

*Marasmius* Fr. (Marasmiaceae, Agaricales) was established by Fries (1835), with *M. rotula* (Scop.) Fr. as the lectotype species (Singer and Smith 1946). Species within the genus are saprophytic and widely distributed worldwide, but show high diversity in tropical and subtropical regions (Singer 1976; Retnowati and Desjardin 2022). More than 700 species have been documented (Bhunjun et al. 2022; Oliveira et al. 2024). *Marasmius oreades* (Bolton) Fr. and *M. heinemannianus* Antonín are recorded as edible (McIlvaine and Macadam 1973; Antonín 1998). Furthermore, *M. oreades* has been demonstrated to exhibit antioxidant, anticancer, antimicrobial, and antibiofilm properties (Shomali et al. 2019). Some *Marasmius* species can cause blight in cash crop (Koo et al. 2025). The full extent of species diversity in this genus remains to be revealed.

Fries (1835) established the earliest infrageneric classification of *Marasmius*, recognizing two sections: (I) *Collybaria*, characterized by a pileus margin initially involute, and (II) *Mycenarii*, distinguished by a margin that is straight from the outset and adnate to the stipe. A third section, *Apus*, encompassing the single sessile species, was subsequently appended. Kühner (1933) expanded the sectional framework to nine. Singer (1951, 1976) further refined the taxonomy, proposing a system of twelve sections that remained authoritative for decades.

Integrating morphological evidence with multi-locus phylogenetic analyses, Oliveira et al. (2024) introduced a hierarchical scheme of subgenus–section–subsection–series. Despite extensive re-definition and re-circumscription prompted by molecular data, *Marasmius* remains a polyphyletic and taxonomically challenging genus. *Marasmius* subgen. *Globulares* J.S. Oliveira, Desjardin & Moncalvo was proposed, characterized by small to large basidiomata, distant to crowded lamellae with several lamellulae, a central to excentric non-insititious stipe and dextrinoid trama (Oliveira et al. 2020). Recently, a new specie occurring gregariously in grasslands has been described in this subgenus (Oguchi and Hosaka 2026). The *Marasmius haematocephalus* species complex and the *Marasmius elegans* species complex have been clarified (Guard et al. 2025, 2026). Although *Marasmius* is a complex and polyphyletic genus, studies on the genus have revealed more than twenty new species in China (Deng and Li 2011; Deng et al. 2011, 2012, 2015, 2017; Chen et al. 2025, Manawasinghe et al. 2025). Most species are placed in the current infrageneric classification system.

The Yunnan-Guizhou Plateau region is rich in biodiversity and serves as a key hotspot for fungal research (Wijayawardene et al. 2021). Previously, the species diversity of macrofungi from the Yunnan-Guizhou Plateau regions have been investigated, and many new species have been discovered from the region (Dong et al. 2024; Ji et al. 2022; Song et al. 2025; Sun et al. 2022; Xu et al. 2025; Zhang et al. 2025). In the research of *Marasmius* in Southwest China, several specimens in subgen. *Globulares* showed unique morphological features and distinct placement in the combined ITS and LSU phylogeny. In this paper, we describe them as three new species based on morphology and multi-locus phylogeny.

## Materials and methods

### Sample collection and morphological study

Fresh basidiomata were photographed in the wild, field notes were taken (Rathnayaka et al. 2025), and the specimens were taken to the laboratory in plastic collection boxes. The sizes of the pileus, stipe and lamellae were measured and recorded. The terms used to describe lamellae spacing refer to the number of lamellae extending from the stipe to the pileus margin and do not include lamellulae, whose spacing is indicated by the number of series present. Color descriptions were obtained according to Kornerup and Wanscher (1978). Samples were dried using an electric dryer at 50 °C and then deposited in the Fungal Herbarium of Guizhou Biological Institute, Guizhou Academy of Sciences, Guizhou, China (HGASMF) and the Cryptogamic Herbarium, Kunming Institute of Botany, Chinese Academy of Sciences (KUN-HKAS). Microscopic features were examined in the laboratory from the dried specimens. All tissues were mounted in 3% aqueous potassium hydroxide (KOH) to rehydrate cells; Melzer’s reagent was used to test for amyloid reaction, and Congo Red reagent to enhance the visibility of specific structures. The sizes of basidiospores, cystidia and pileipellis cells were measured under a 100× objective. For spore measurement, the abbreviation Q refers to the length/width ratio, and Qm refers to the mean of Q values. Values in square brackets are standard deviations [SD] for mean measurements of length, width and Qm values. N = number of spores measured per specimen.

### DNA extraction, polymerase chain reaction (PCR) amplification, and sequencing

Genomic DNA was extracted from dried specimens using the Ezup Column Fungi Genomic DNA Purification Kit and stored at -20 °C. The nuclear ribosomal internal transcribed spacer (nrITS) and nuclear ribosomal large subunit (nrLSU) were amplified using the primer pairs ITS1F/ITS4 and LR0R/LR5, respectively (White et al. 1990, Hopple and Vilgalys 1999). PCR amplification was performed in a total volume of 25 μL, containing 1 μL template DNA, 9.5 μL distilled water, 1 μL of each primer, and 12.5 μL 2 × PCR mix (DreamTaq^tm^ Green PCR Master Mix, Fermentas). The PCR procedure amplification was as follows: pre-denaturation at 94 °C for 5 min, 35 cycles of denaturation at 94 °C for 30 s, annealing at 58 °C (for nrITS)/58 °C (for nrLSU) for 40 s, extension at 72 °C for 1 min, and final extension at 72 °C for 10 min. After the amplification products were tested by agarose gel electrophoresis, the PCR products were sent to Beijing BGI Co., Ltd. (Guangzhou, China) for sequencing. Bidirectional sequences were checked and assembled using Sequencher version 4.1.2. All newly generated sequences were submitted to GenBank.

### Phylogenetic analyses

The new species sequences were analyzed using BLAST against the NCBI database (https://blast.ncbi.nlm.nih.gov), and only sequences with >90% similarity were selected. The nrITS and nrLSU datasets were concatenated for phylogenetic reconstruction. The newly generated sequences and the sequences of ser. *luteolus*, *purpureostriatus*, and *wynneae* from Oliveira et al. (2024) were selected to construct the combined dataset. Multiple sequence alignment was performed using MAFFT v.7 (https://mafft.cbrc.jp/alignment/server/index.html), and poorly aligned regions were removed with TrimAl v.1.3. A gap threshold of 0.5 was applied for both ITS and nrLSU fragments. The trimmed sequences were assembled into a combined dataset in the order of ITS-nrLSU using SequenceMatrix v.1.8.

Maximum likelihood (ML) analysis was performed on the CIPRES Science Gateway platform (http://www.phylo.org/) using RAxML HPC2 v.8.2.1, under the GTR+G+I model with 1000 bootstrap replicates to assess branch support. Bayesian inference (BI) analysis was performed using MrBayes v.3.2, with Markov chain Monte Carlo (MCMC) sampling to calculate posterior probabilities (PP). MrModeltest v.2.3 was used to determine the best-fit nucleotide substitution model for each gene under the Akaike information criterion (AIC). The GTR+I+G model was selected as the optimal substitution model for both the ITS and nrLSU datasets. Four independent Markov chains were run simultaneously for 1,815,000 generations, with trees sampled every 100 generations. The first 25% of sampled trees were discarded as burn-in, and convergence was confirmed when the average standard deviation of split frequencies fell below 0.01, and all effective sample sizes (ESS) exceeded 200. The resulting phylogenetic tree was visualized in FigTree v.1.4.4 and further edited using Adobe Illustrator CC 2018 (Adobe Systems, USA) (Table 1).

**Table 1.** The species, specimens, and GenBank accession numbers of ITS & LSU sequences analyzed in this study. Names in bold represent newly described species in this study. All type specimens are highlighted with a “T”. The symbol “–” denotes no available data.

**Species**	**Voucher**	**GenBank Accession No**.		**References**
		**ITS**	**nrLSU**	
*Collybia fissipes*	16285	JF908342	–	Oliveira et al. (2024)
*Marasmius aff. Camerunensis*	KUM 60134	FJ431229	–	Oliveira et al. (2024)
*M. albogriseus*	JLF4827	MH090925	–	Oliveira et al. (2024)
*M. albopurpureus* T	GDGM57201	KP127674	KP127676	Oliveira et al. (2024)
*M. albopurpureus*	GDGM57089	KP127675	–	Oliveira et al. (2024)
*M. angustilamellatus* T	TYS524	FJ431219	–	Oliveira et al. (2024)
*M. angustilamellatus*	TYS437	FJ431218	–	Oliveira et al. (2024)
*M. araneocephalus* T	NW358	EU935540	–	Oliveira et al. (2024)
*M. auratus*	NW076	EU935501	–	Oliveira et al. (2024)
*M. auratus* T	NW175	EU935502	–	Oliveira et al. (2024)
*M. bekolacongoli*	Lockwood 2131638	KX148982	–	Oliveira et al. (2024)
*M. campestris*	HKAS80858	KJ126767	KJ126769	Oliveira et al. (2024)
*M. campestris* T	HKAS80857	KJ126766	KJ126768	Oliveira et al. (2024)
*M. centrocinnamomeus* T	SFC20180704-02	OP730947	–	Liu et al. (2024)
*M. centrocinnamomeus*	SFC20140703-03	OP730948	–	Liu et al. (2024)
*M. coasiaticus* T	JO323	ON502680	ON502747	Oliveira et al. (2024)
*M. decipiens*	DED3612	OR636642	OR656967	Oliveira et al. (2024)
*M. delectans*	DED4146	OR636643	OR656968	Oliveira et al. (2024)
*M. delectans*	Mushroom Observer 283976	MK607539	–	Oliveira et al. (2024)
*M. elegans*	LNP598	PP354943	–	GenBank
*M. elegans*	JAC11770	PP407531	–	GenBank
*M. fiardii*	PR-910	OM238177	–	Oliveira et al. (2024)
*M. fissuratus* T	GDGM 30013	KC191812	–	Oliveira et al. (2024)
***M. flavosulcatus* T**	**HGASMF01-32728**	**PZ288149**	**PZ292008**	**This study**
***M. flavosulcatus***	**HGASMF01-28414**	**PZ289855**	**PZ292016**	**This study**
***M. flavosulcatus***	**HGASMF01-31659**	**PZ288156**	**–**	**This study**
***M. flavosulcatus***	**HKAS154778**	**PZ288155**	**PZ292015**	**This study**
*M. florideus*	TYS480	FJ431240	–	Oliveira et al. (2024)
*M. galbinus* T	GDGM 27251	HQ709445	–	Oliveira et al. (2024)
*M. grandiviridis* T	NW152	OR636648	OR656977	Oliveira et al. (2024)
***M. griseobrunneus* T**	**HKAS154776**	**PZ289854**	**PZ292014**	**This study**
***M. griseobrunneus***	**HKAS154775**	**PZ288153**	**–**	**This study**
***M. griseobrunneus***	**HKAS154777**	**PZ289852**	**PZ292013**	**This study**
***M. griseobrunneus***	**HGASMF01-29990**	**PZ289853**	**PZ292007**	**This study**
***M. griseobrunneus***	**HGASMF01-31535**	**PZ288154**	**–**	**This study**
*M. indojasminodorus*	AKD139/2015	KY785171	KY785173	Oliveira et al. (2024)
*M. indojasminodorus* T	AKD135/2015	KY785172	KY785174	Oliveira et al. (2024)
*M. indopurpureostriatus* T	CAL KD 14-001	NR_154185	KT004443	Oliveira et al. (2024)
*M. inthanonensis* T	NW353	EU935514	–	Oliveira et al. (2024)
*M. jasminodorus*	GCMCC17072	MK656317	–	Oliveira et al. (2024)
*M. jasminodorus*	NW414	EU935515	–	Oliveira et al. (2024)
*M. laranja* T	DED 8231	KX953748	–	Oliveira et al. (2024)
*M. laticlavatus*	NW412	EU643511	–	Oliveira et al. (2024)
*M. laticlavatus*	NW293	EU643512	–	Oliveira et al. (2024)
*M. luteolus*	NW138	EU935506	–	Oliveira et al. (2024)
*M. luteolus*	NW304	EU935507	–	Oliveira et al. (2024)
*M. maximus*	BRNM 714571	FJ904977	FJ904959	Oliveira et al. (2024)
*M. maximus*	BRNM 714570	FJ904976	FJ904958	Oliveira et al. (2024)
*M. maximus*	B6	JX434662	–	Oliveira et al. (2024)
*M. maximus*	NS16081406	MN523279	–	Oliveira et al. (2024)
*M. maximus*	MHHNU 8461	MK250944	–	Oliveira et al. (2024)
*M. megistus*	DED 8230	KX953750	–	Oliveira et al. (2024)
*M. megistus*	JES 163	KX148992	–	Oliveira et al. (2024)
*M. megistus*	Lockwood 2132155	KX148993	–	Oliveira et al. (2024)
*M. midnapurensis* T	CUH AMT002	KY785179	–	Oliveira et al. (2024)
*M. midnapurensis*	CAL1523	MF189041	–	Oliveira et al. (2024)
*M. mokfaensis*	DED7726	OR636658	OR656997	Oliveira et al. (2024)
*M. mokfaensis*	NW1433	MW426457	–	Oliveira et al. (2024)
*M. multicystidiatus* T	F2015002	PP354939	PP354931	Oliveira et al. (2024)
*M. neooreades* T	TNS F-84727	PX091517	PX090884	Oguchi and Hosaka (2026)
*M. neooreades*	TNS F-84894	PX091518	PX090885	Oguchi and Hosaka (2026)
*M. nivicola*	BRNM 714575	FJ904972	FJ904954	Oliveira et al. (2024)
*M. nivicola* T	KPM-NC 0006038	FJ904973	FJ904955	Oliveira et al. (2024)
*M. nivicola*	BRNM 714572	FJ904970	FJ904952	Oliveira et al. (2024)
*M. occultatiformis*	MHHNU30835	MK388150	–	Oliveira et al. (2024)
*M. occultatiformis*	JBRI-M21-075	PQ613368	–	GenBank
*M. ochroleucus*	LE295978	KF912952	KF896249	Oliveira et al. (2024)
*M. ochroleucus*	HMJAU63586	OR364586	–	Oliveira et al. (2024)
*M. ochroleucus*	NW299	EU935503	–	Oliveira et al. (2024)
*M. ochropoides* T	TYS384	FJ431263	–	Oliveira et al. (2024)
*M. ochropoides*	KLU-M89	NR154150	–	Oliveira et al. (2024)
*M. odoratus* T	CAL:1264	KT180332	–	Oliveira et al. (2024)
*M. oreades*	WA0000071064	MK028920	–	Oliveira et al. (2024)
*M. oreades*	BAFC4558	KY366495	–	Oliveira et al. (2024)
*M. oreades*	UBC F-23953	MF955189	–	Oliveira et al. (2024)
*M. oreades*	PBM 2701	DQ490641	–	Oliveira et al. (2024)
*M. oreades*	NN055694	JN943604	JN941144	Oliveira et al. (2024)
*M. oreades*	ASIS21388	KF668290	–	Oliveira et al. (2024)
*M. oreades*	ZRL2015086	LT716048	KY418864	Oliveira et al. (2024)
*M. oreades*	HFJAU0311	MN258668	–	Oliveira et al. (2024)
*M. pseudopellucidus*	NW186	EU935504	–	Oliveira et al. (2024)
*M. pseudopellucidus* T	NW305	EU935505	–	Oliveira et al. (2024)
*M. pseudopurpureostriatus* T	NW286	OR636672	OR657016	Oliveira et al. (2024)
*M. puerariae*	Kirschner 2139	JX470333	JX470332	Oliveira et al. (2024)
*M. purpureostriatus*	NW158	EU935539	–	Oliveira et al. (2024)
*M. purpureostriatus*	NW318	EU935538	–	Oliveira et al. (2024)
*M. purpureostriatus*	BRNM 714566	FJ904978	FJ904960	Oliveira et al. (2024)
*M. purpureostriatus*	JBRI-M23-047	PQ613369	–	GenBank
*M. rubicundus* T	SP 445549	NR_184981	NG_243114	Oliveira et al. (2024)
*M. rubicundus*	JO246	ON502664	ON502725	Oliveira et al. (2024)
*Marasmius* sp.	KA-2017 SURJIT-02	MF189075	–	Oliveira et al. (2024)
*Marasmius* sp.	KA-2017 SURJIT-01	MF189074	–	Oliveira et al. (2024)
*Marasmius* sp.	HKAS 148679	PX353111	–	Oliveira et al. (2024)
*Marasmius* sp.	GMB2014 MEL:2382888	KP012759	–	Oliveira et al. (2024)
*Marasmius* sp.	KUS-F32028	PV400564	PV391003	GenBank
*Marasmius* sp.	KUS-F31984	PV390996	PV390997	GenBank
*Marasmius* sp.	KUS-F34275	PV391002	PV391004	GenBank
*M. strobiluriformis* T	BRNM714914	GU266263	GU266270	Oliveira et al. (2024)
*M. strobiluriformis*	BRNM714915	GU266264	GU266271	Oliveira et al. (2024)
***M. variegatus* T**	**HKAS154773**	**PZ288151**	**PZ292011**	**This study**
***M. variegatus***	**HKAS154774**	**PZ288152**	**PZ292012**	**This study**
***M. variegatus***	**HKAS135842**	**PZ288150**	**PZ292010**	**This study**
*M. wynneae*	HCCN-G86	FJ904979	FJ904961	Oliveira et al. (2024)
*M. wynneae*	DM1048	MT644909	–	Oliveira et al. (2024)

## Results

### Phylogenetic analyses

**Figure 1.**
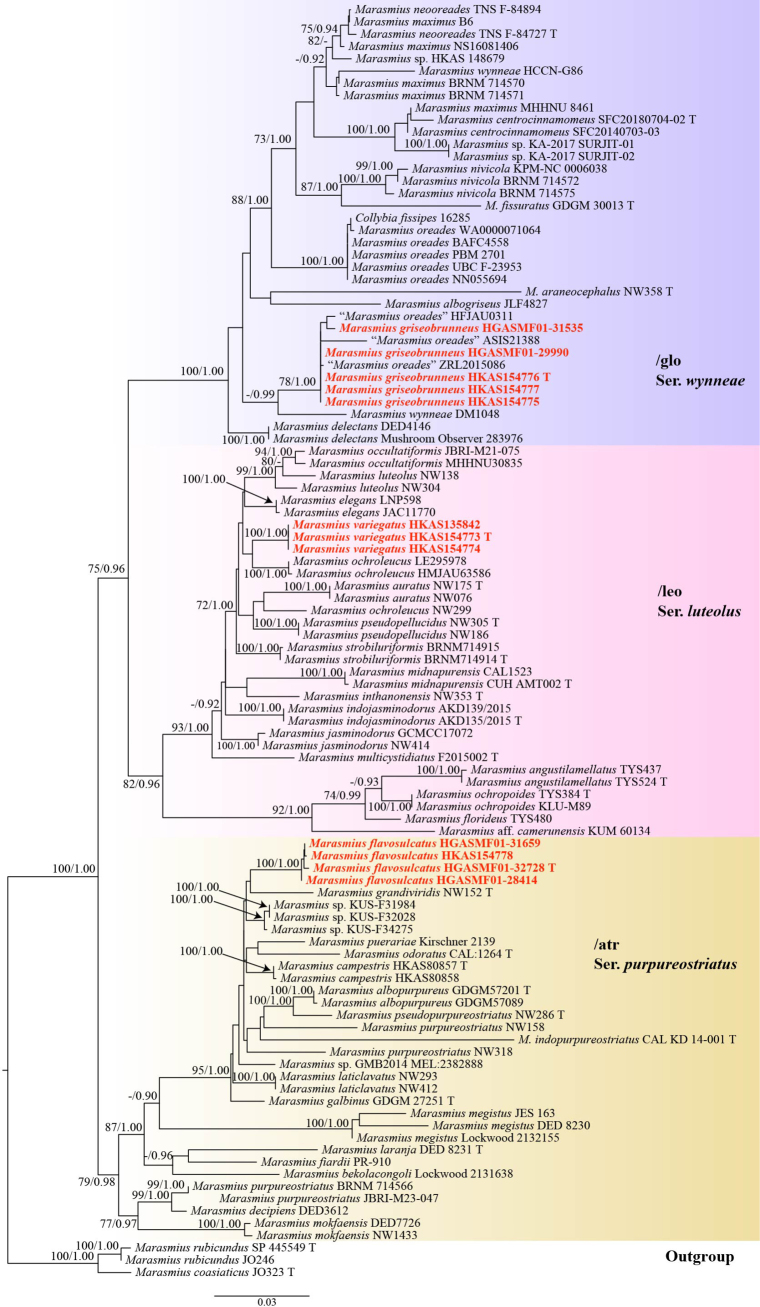
Phylogram of *Marasmius* generated by CIPRES analysis based on sequences of ITS + LSU. It was rooted with *M. rubicundus* and *M. coasiaticus*. ML bootstrap proportions (ML-BP) ≥ 70% and Bayesian posterior probabilities (BI-PP) ≥ 0.90 are indicated above the nodes. The new taxa are marked in bold red. “-” indicates that the support value was less than the respective threshold. “T” indicates that the type specimens.

A total of 1,573 characters from 104 taxa were used in phylogenetic analyses (ITS 685 bp; LSU 888 bp), of which 1,097 were constant, 374 were parsimony-informative, and 102 were singletons. The best-fit ML model was GTR+G+I. Phylogenetic results are present in Fig. 1. The three new species each formed distinct, well-supported lineages. *Marasmius flavosulcatus* belongs to /purpureostriatus clade and groups with *M. grandiviridis*. *Marasmius griseobrunneus* belongs to /wynneae clade and groups together with *M. wynneae*. The specimens labeled as “*Marasmius oreades*” were also nested within the clade of the new species, thus, the vouchers HFJAU0311, ASIS21388 and ZRL2015086 should be identified as *M. griseobrunneus*. *Marasmius variegatus* belongs to /*luteolli* clade and is closely related to *M. ochroleucus*.

### Taxonomy

#### Marasmius flavosulcatus

Taxon classificationFungiAgaricalesMarasmiaceae

Chun Y. Deng sp. nov.

50F82745-68D1-52AE-A5F5-05FCC6668C0D

Fungal Names: FN 573723

Fig. 2

##### Chinese name.

黄沟纹小皮伞 (huang gou wen xiao pi san).

**Figure 2.**
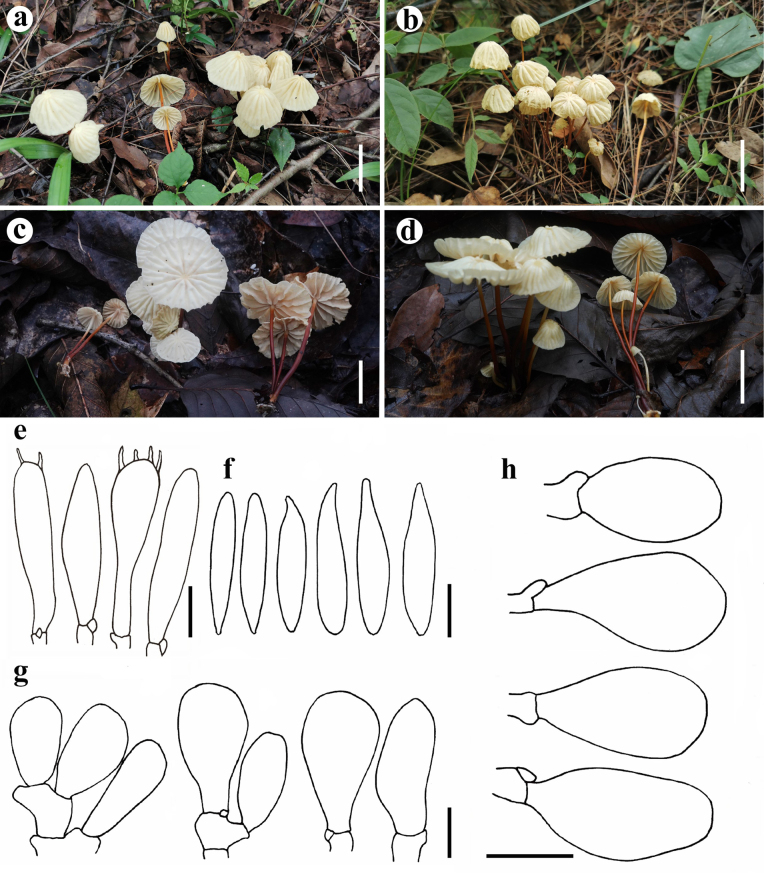
Morphological structures of *Marasmius flavosulcatus* (HGASMF01-32728, holotype). **a–d**. Basidiomata; **e**. Basidia; **f**. Basidiospores; **g**. pileipellis; **h**. Cheilocystidia. Bar: 2 cm (**a–d**); 10 μm (**e–h**).

##### Etymology.

*Flavosulcatus* refers to the yellow, plicate pileus.

##### Holotype.

China • Guizhou province: Tongren City, Guizhou Mayanghe National Nature Reserve, at 28°39'13.00"N, 108°13'8.67"E, alt. 667.82 m, 23 Sep. 2025, HGASMF01-32728 (DCY10537).

##### Diagnosis.

*Marasmius flavosulcatus* is characterized by a plicate, pale-yellow pileus, a long reddish-yellow stipe, large basidiospores, globulare, hymeniform pilepellis, and the absence of pleurocystidia and caulocystidia. It differs from *Marasmius grandiviridis* by small yellow basidiomes lacking olive coloration, more regularly clavate, bulbous cheilocystidia (Wannathes et al. 2009a).

##### Description.

***Basidiomata*** medium to large. ***Pileus*** 1–4 cm broad, hemispherical when young, plano-convex with age, umbilicate, yellowish white (3A2, 4A2), deeply sulcate and striate. ***Lamellae*** adnexed, subdistant, L = 9–13 (l = 0–1), 3–5 mm broad, thick, non-marginate, non-intervenose, yellowish white (3A2); edge smooth and entire, ***Context*** thin, concolorous with pileus. ***Stipe*** 50–95 × 1–2 mm, central, cylindrical, hollow; surface dry, non-insititious, apex whitish, becoming yellowish white (3A2), light orange (6A5), greyish orange (6B5). ***Taste*** and ***odor*** not distinctive.

***Basidiospores*** 27–34 × 3.8–5.4 µm, Q = 5.4–8.5, on average 29.45 [± 1.82 SD] × 4.73 [± 0.45 SD] µm, Qm = 6.29 [± 0.91SD], N = 40 from 4 collections, elongate clavate to fusoid, often curved in profile, smooth, hyaline, inamyloid, thin-walled. ***Basidia*** 30–51 × 7–11 µm, clavate, 2- or 4-spored. ***Cheilocystidia*** abundant, 17–30 × 7–15 µm, pyriform, clavate. ***Pileipellis*** a hymeniform layer of Globulares-type cells, 14–28 × 7–15 µm, broadly clavate to pyriform, hyaline, inamyloid, thin- to thick-walled, non-gelatinous. ***Lamellar trama*** regular; hyphae 2.5–10 μm diam., cylindrical, hyaline, dextrinoid, thin-walled. ***Stipitipellis hyphae*** 4–8 (–10) µm diam., subparallel, cylindrical, yellow to greenish yellow, smooth, dextrinoid. ***Caulocystidia*** absent. ***Clamp connections*** present in all tissues.

##### Habitat.

Gregarious on humus in forest.

##### Additional collection examined.

China • Guizhou Province: Guiyang City, 26°37'16.118"N, 106°42'24.825"E, alt. 1290.15 m, 21 Jun. 2024, HKAS 154778 (DCY8225, paratype); ibid, HGASMF01-28414 (DCY8213); China • Guizhou province: Tongren City, Guizhou Mayanghe National Nature Reserve, 28°39'24.74"N, 108°13'4.63"E, alt. 643.30 m, 7 Jun. 2025, HGASMF01-31659 (DCY10011).

##### Notes.

A basepair comparison between the holotype sequence of the new species and the closely related species *Marasmius grandiviridis* Wannathes, Desjardin & Lumyongs, revealed 92.82% identity with 45 substitutions/indels in ITS, and 98.53% identity with 13 substitutions/indels in LSU, supporting its recognition as a distinct species. Based on the phylogenetic tree, the new species is nested in subg. *Globulares* ser. *Purpureostriatus* (Oliveira et al. 2020; Oliveira et al. 2024). Compared to the medium- to large-sized basidiomata and sulcate pileus, the new species is morphologically similar to *M. bekolacongoli* Beeli and *M. campestris* N.K. Zeng, Zhi Q. Liang & M.S. Su, *Marasmius galbinus* T.H. Li & Chun Y. Deng and *M. grandiviridis*. *Marasmius bekolacongoli* has a red or grayish red pileus, with violet to dark violaceous tints in suici, and is described as having smaller basidiospores, 20 × 4.5 µm (Antonín 2007). *Marasmius campestris* differs by its purple-tinged pileus, shorter stipe, and presence of caulocystidia (Liang et al. 2017). *Marasmius galbinus* differs by its smaller, paler, conical pileus and shorter basidiospores (14–16 *vs*. 27–34 μm) (Deng and Li 2011). *Marasmius grandiviridis* has a larger, yellowish-green pileus with darker olive-green plicate and irregularly clavate to lageniform-mucronate cheilocystidia (Wannathes et al. 2009a).

#### Marasmius griseobrunneus

Taxon classificationFungiAgaricalesMarasmiaceae

Chun Y. Deng sp. nov.

0B6C7BC5-B797-5DE4-85D8-00D412214AFF

Fungal Names: FN 573724

Fig. 3

##### Chinese name.

灰褐小皮伞 (hui he xiao pi san).

**Figure 3.**
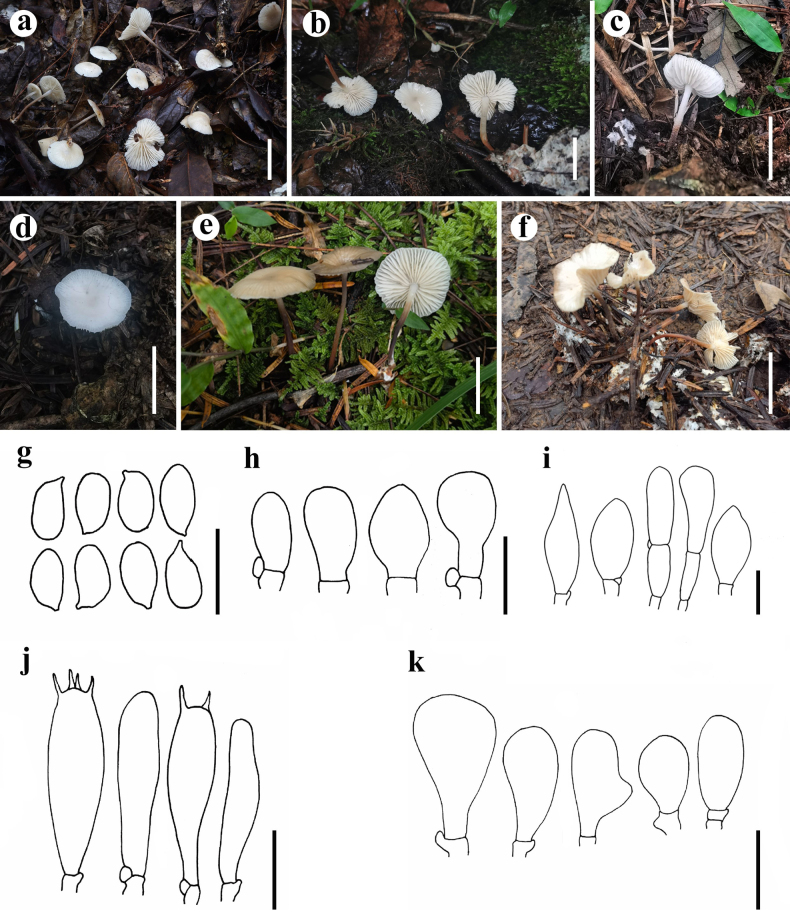
Morphological structures of *Marasmius griseobrunneus* (HKAS154776, holotype). **a–f**. Basidiomata; **g**. Basidiospores; **h**. Cheilocystidia; **i**. Caulocystidia; **j**. Basidia; **k**. Pileipellis. Bar: 2 cm (**a–f**); 10 μm (**g–k**).

##### Etymology.

“*griseobrunneus*” refers to its pileus becoming grayish brown when mature.

##### Holotype.

China • Yunnan Province: Kunming City, Heilongtan Park, 25°08'06.76"N, 102°44'29.57"E, alt. 1949.09 m. 22 Oct. 2025, collected by Chunying Deng, HKAS154776 (DCY10741).

##### Diagnosis.

*Marasmius griseobrunneus* is characterized by gymnopoid basidiomata, small to medium-sized, applanate, occasionally shallowly infundibuliform, grayish brown pileus, medium-sized basidiospores, and pileipellis composed of hymeniform Globulares-type cells. It differs from *Marasmius wynneae* by a smaller pileus (3 cm vs. 5 cm), subdistant lamellae, an olivaceous brown pileus, and dense caulocystidia.

##### Description.

***Basidiomata*** small to medium-sized. ***Pileus*** 1–3 cm, broadly convex when young, applanate at maturity, sometimes centrally depressed to form a shallow funnel, white to cream when young, becoming orange-gray (6B2) to grayish brown (6D3), glabrous to weakly striate, slightly hygrophanous at the margin. ***Lamellae*** adnate, subdistant, L = 15–18(l = 2–4), 1–2 mm broad, white, reticulate or intervenose. ***Context*** white, thin. ***Stipe*** 35–40 × 2–5 mm, central, cylindrical, tapering toward the base, apex white, becoming reddish brown (8D5, 8F5), hollow, fibrous, covered with white furfuraceous scales. ***Base mycelium*** white. ***Taste*** and ***odor*** not distinctive.

***Basidiospores*** 6.3–7.8 × 3.2–4.4 µm, Q = 1.5–2.3, mean 7.06 [± 0.42 SD] × 3.91 [± 0.34 SD] µm, Qm = 1.82 [± 0.22 SD], N = 60 from three collections, ellipsoid, thin-walled, smooth, inamyloid. ***Basidia*** 24–35 × 4–6 µm, clavate, 2- or 4-spored. ***Basidioles*** fusoid, cylindrical to clavate. ***Cheilocystidia*** common, 14–21 × 3.5–12 µm, broadly clavate to pyriform or subcylindrical, thin-walled. ***Pleurocystidia*** absent. ***Pileipellis*** a hymeniform layer of Globulares-type cells; terminal elements 13–32 × 9.3–15 μm, broadly clavate to pyriform or subcylindrical, thin-walled. ***Lamellae trama*** interwoven, with hyphae 3.5–7 μm in diam, hyaline, dextrinoid, thin-walled. ***Stipitipellis*** a cutis composed of cylindrical hyphae 3–7 μm wide, parallel, smooth. ***Caulocystidia*** cluster or single with clavate, subclavate, globulares to pyriform cells, 10–31 × 6.8–11 µm. ***Clamp connections*** present.

##### Habitat.

Scattered to gregarious on humus in mixed coniferous and broad-leaved forests.

##### Additional collection examined.

China • Yunnan Province: Kunming City, Heilongtan Park, 25°08'06.76"N, 102°44'29.57"E, alt. 1949.09 m. 22 Oct. 2025, collected by Chunying Deng, HKAS154775 (DCY10692); • ibid, Jindian Park, 25°05'10.08"N, 102°46'16.08"E, alt. 1999.84 m. 23 Oct. 2025, HKAS 154777 (DCY10758, paratype). China • Guizhou Province: Tongren City, Guizhou Mayanghe National Nature Reserve, 28°48'54.60"N, 108°10'51.96"E, alt. 1027.13 m, 31 May 2025, HGASMF01-31535 (DCY9854). China • Guizhou Province: Zunyi City, Guizhou Xishui National Nature Reserve, 28°09'54.72"N, 105°54'31.92"E, alt. 735.29 m, 21 Oct. 2024, HGASMF01-29990 (DCY8989).

##### Notes.

A basepair comparison between the holotype sequence of the new species and the closely related species *Marasmius wynneae* Berk. & Broome (specimen voucher: DMS-9344190), revealed 92.62% identity with 52 substitutions/indels in ITS, and 98.94% identity with 9 substitutions/indels in LSU, supporting its recognition as a distinct species. Based on molecular phylogenetic and morphological analyses, *Marasmius griseobrunneus* belongs to subg. *Globulares* ser. *Wynnearum* (Oliveira et al. 2020; Oliveira et al. 2024). Within this series, *Marasmius wynneae*, *M. nivicola* and *M. albogriseus* (Peck) Singer are morphologically similar to the new species. However, *M. wynneae* lacks caulocystidia (Antonín et al. 2010a). *Marasmius nivicola* differs by a whitish pileus, crowded and distinctly intervenose lamellae, a solid mycelium mat around the stipe base, and slender cheilocystidia (Takahashi 2000). *Marasmius albogriseus* is distinguished by the lighter colored pileus, a well-developed tomentum at the stipe base, and gregarious and caespitose habitat (Halling et al. 1985).

#### Marasmius variegatus

Taxon classificationFungiAgaricalesMarasmiaceae

Chun Y. Deng & Qi Zhao sp. nov.

A0D648F9-E922-5739-BB0F-4AE4E85F5210

Fungal Names: FN 573725

Fig. 4

##### Chinese name.

杂色小皮伞 (za se xiao pi san).

**Figure 4.**
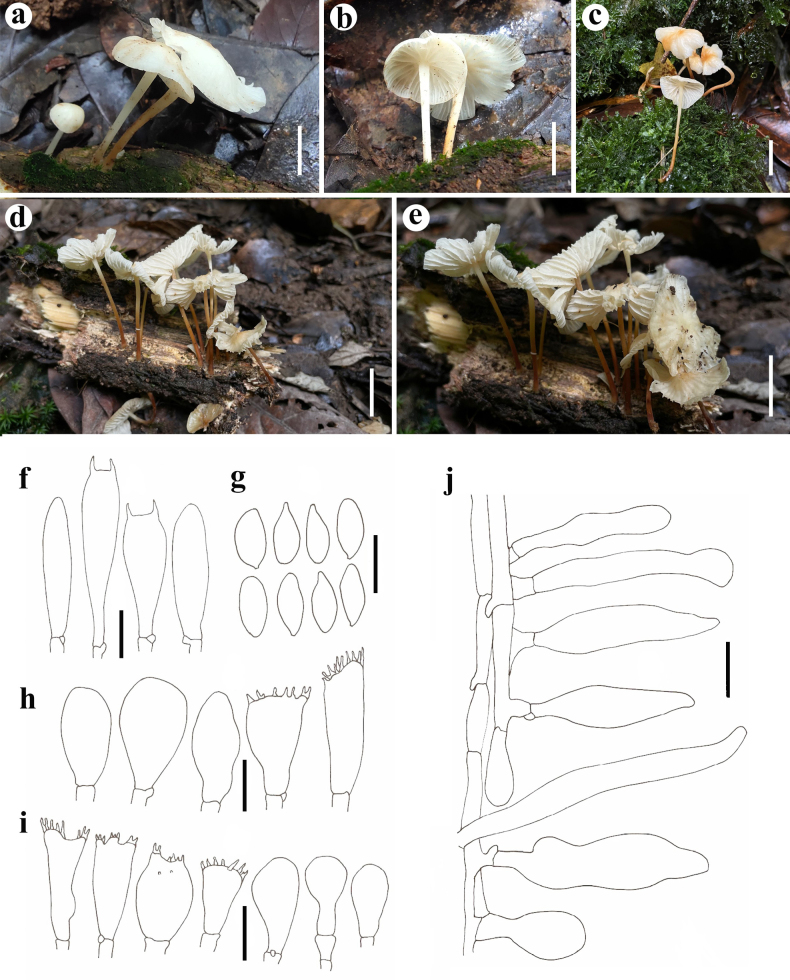
Morphological structures of *Marasmius variegatus* (HKAS154773, holotype). **a–e**. Basidiomata; **f**. Basidia; **g**. Basidiospores; **h**. Cheilocystidia; **i**. Pileipellis; **j**. Caulocystidia. Bar: 1 cm (**a–e**); 10 μm (**f–j**).

##### Etymology.

“*variegatus*” refers to its white pileus mottled with irregular yellowish-brown areas.

##### Holotype.

China • Yunnan Province: Puer City, Jingdong Yi Autonomous County, Ailao Mountains, 24°32'05"N, 101°01'12"E, alt. 2512 m, 25 Aug. 2025, collected by Tai-Shun Li, HKAS 154773 (LTS131)

##### Diagnosis.

*Marasmius variegatus* is characterized by a pileus with uneven coloration, subdistant, narrow and branched lamellae, a stiff stipe, medium-sized basidiospores, pileipellis and cheilocystidia composed of two cell types, and relatively long caulocystidia. It differs from *M. ochroleucus* by its crowed and close lamellae, and the presence of two-types of cells in both cheilocystidia and pileipellis.

##### Description.

***Basidiomata*** medium-sized. ***Pileus*** 0.8–3 cm, broadly convex to applanate, white with light orange (5A5), and grayish orange patches (5B4, 5B6), hygrophanous, faintly striate, margin recurved with age. ***Lamellae*** adnate, subdistant, L = 11–12 (l = 2–5), white, narrow, frequently forked. ***Stipe*** 40–60 × 1–1.5 mm, central, cylindrical, white when young, gradually becoming grayish orange (5B6), golden yellow (5B7, 6B7), reddish golden (6C7), base with thick, robust mycelium, brownish orange (6C8).

***Basidiospores*** 9–12 × 4–5.3 µm, Q = 1.8–2.8, mean 10.28 [± 0.98 SD] × 4.66 [± 0.34 SD] µm, Qm = 2.22 [± 0.28 SD], N = 60 from three collections, ellipsoid, thin-walled, smooth, inamyloid. ***Basidia*** 27–35 × 5–7.8 µm, clavate, 2- or 4-spored. ***Basidioles*** fusoid, cylindrical to clavate, hyaline, thin-walled, inamyloid. ***Pleurocystidia*** absent. ***Cheilocystidia*** similar to the cells of the pileipellis: 1) Siccus-type cells, hyaline when grouped; main body 12–28 × 6–15 µm, clavate to pyriform, thin-walled, hyaline, inamyloid; diverticula or setulae apical, short and somewhat divergent, 1–2.5 × 0.6–1 µm, verruciform to cylindrical, digitiform, regular in outline or a little monilioid, many times vesiculose-rounded or balloon-shaped, abundant to scarce, solid, apex obtuse and rounded; 2) Globulares-type cells, 10–24 × 6–13 µm, more thin-walled. ***Lamellae trama*** inamyloid, irregular, hyphae cylindrical, 1.5–5 µm diam., regular in outline, hyaline, branched, smooth, thin-walled. ***Pileipellis*** hymeniform, composed of two cellular types: 1) Siccus-type broom cells, main body 15–24 × 6–12 µm, pyriform to cylindrical-clavate, hyaline, thin-walled; setulae apical, erect, elongate to very short, 1–3 × 1–2 µm, cylindrical, digitiform or verruciform, regular or irregular in outline, abundant or scarce, solid or with lumen, apex acute to obtuse; 2) Globulares-type smooth cells, more numerous than the first type at the mature basidiomata, 16.3–26.3 × 12.5–17.5 µm, pyriform or balloon-shaped, vesiculose to clavate, or irregular in outline, hyaline, smooth or apical portion with thicker walls, dextrinoid. ***Caulocystidia*** 15–70 × 5–10 µm, clavate, fusoid, irregular in outline, obtuse, hyaline, smooth, inamyloid. ***Clamp connections*** present in all tissues.

##### Habitat.

Solitary or gregarious on well-rotted bark or wood.

##### Additional collection examined.

China • Yunnan Province: Puer City, Jingdong Yi Autonomous County, Ailao Mountains, 24°32'26"N, 101°01'40"E, alt. 2603 m, 25 Aug. 2022, collected by Jin-Rong Lu, KHAS135842 (LJR-210); • ibid, 24°32'11"N, 01°01'56"E, alt. 2606 m, 18 Jul. 2024, Tai-Shun Li, HKAS154774 (LTS313, paratype).

##### Notes.

A basepair comparison between the holotype sequence of the new species and the closely related species *Marasmius ochroleucus* Desjardin & E. Horak (specimen voucher: LE295978), revealed 96.17% identity with 26 substitutions/indels in ITS, and 98.95% identity with 9 substitutions/indels in LSU, supporting its recognition as a distinct species. Based on molecular phylogenetic evidence, *Marasmius variegatus* belongs to subg. *Globulares* ser. *Luteoli* (Oliveira et al. 2020; Oliveira et al. 2024). The new species forms an independent clade and is the sister taxon of *M. ochroleucus*. The latter species has only one type of broom cell in the pileipellis and cheilocystidia, as well as densely crowded lamellae. Comparing the cream, hygrophanous pileus, the new species is similar to *Marasmius pseudopellucidus* Wannathes and *M. strobiluriformis* Antonin, R. Ryoo & H.D. *M. pseudopellucidus* has larger basidiospores (Xm = 11 × 4.1 µm), grows gregariously on bamboo debris and possesses only siccus-type cheilocystidia (Wannathes et al. 2009b). *Marasmius strobiluriformis* differs by the pure white, smaller pileus, more crowded lamellae, and rostrate cheilocystidia (Antonín et al. 2010b).

## Discussion

Oliveira et al. (2020) addressed the non-monophyletic nature of the infrageneric classification within the sect. *Globulares* as defined by Antonín and Noordeloos (2010). Based on the phylogenetic analysis of ITS and LSU sequences, they elevated several of Singer’s (1976) original stripes to the rank of series. In our phylogenetic reconstruction, the three new species are placed within subgen. *Globulares*, sect. *Globulares*. Specifically, they affiliate with subsect. *Leonini* ser. *Luteoli*, subsect. *Atrorubentes* ser. *Purpureostriati*, and subsect. *Globulares* ser. *Wynnearum*, respectively. Each new species forms a well-supported, distinct terminal clade within one of these three series, thereby reinforcing the validity of this revised sectional subdivision.

*Marasmius flavosulcatus* exhibits the consistent morphological characteristics of ser. *Purpureostriati*, which include a striate pileus, long-ellipsoid basidiospores (20–35 µm), absent pleurocystidia, a globular-type hymeniform pilepellis, and present caulocystidia (Oliveira et al. 2020). *Marasmius grandiviridis*, reported from Mauritius, was found to be a close relative of *M. flavosulcatus*; it differs from our new species by its larger (37–88 mm) and darker, olive-green plicate pileus (Wannathes et al. 2009a). *Marasmius griseobrunneus* shows the morphological features of ser. *Wynnearum*: a fleshy pileus, anastomosed or reticulate lamellae, a relatively robust stipe, and ovoid to ellipsoid basidiospores (6–10.3 µm in length). In the phylogenetic analyses, *Marasmius griseobrunneus* formed a clade with two Chinese sequences (ZRL2015086 and HFJAU0311) and one Korean sequence (ASISI1388). BLAST comparisons of the ITS region revealed high sequence similarity between the holotype of *Marasmius griseobrunneus* (DCY10741) and these sequences (100% for ZRL2015086, 99% for HFJAU0311, and 98% for ASIS21388).

*Marasmius variegatus* exhibits traits characteristic of ser. *Luteoli*: pileus glabrous or striate, lamellae of unequal lengths, stipe pruinose, pubescent to hispidulous, and basidiospores medium-sized (8–14 µm long). However, the cheilocystidia and pileipellis cells comprise two types of cells—a feature not previously reported in other species of this series.

Guard et al. (2026) established ser. *Multicystidiatus* when studying the *Marasmius elegans* species complex within ser. *Luteoli*. Oguchi and Hosaka (2026) reported a new species belonging to ser. *Wynnearum*. This indicates that the species diversity of the genus *Marasmius* and its infrageneric subdivision system still requires further investigation and refinement. This study confirms that subgen. *Globulares* represents a rapidly radiating lineage.

The recorded finding of three new *Marasmius* species with morphological and molecular date enriches our understanding of species diversity within the genus, provides valuable insights for taxonomic revision and helps clarify species boundaries among closely related taxa.

## Supplementary Material

XML Treatment for Marasmius flavosulcatus

XML Treatment for Marasmius griseobrunneus

XML Treatment for Marasmius variegatus

## Additional information

### Artificial Intelligence (AI) use

The authors accept full responsibility for the content of the manuscript, including the disclosure of any use of AI.

No AI tools were used in the preparation of this manuscript.

### Author ORCIDs

C.-Y. Deng https://orcid.org/0000-0002-0960-0948

T.-S. Li https://orcid.org/0000-0001-8697-1630

Z. Xiang https://orcid.org/0009-0000-5548-2842

Q. Zhao https://orcid.org/0000-0001-8169-0573

### Data availability

All of the data that support the findings of this study are available in the main text.
